# Our Exchanges

**Published:** 1873-01

**Authors:** 


*Our Exchanges*

Will do us the favor to forward their publications to the SOUTHERN MEDICAL RECORD. A few medical journals, from inadvertency, we suspect, have never sent us an exchange. We hope they will have the RECORD placed on their exchange list.

